# Spin-selective transport in a correlated double quantum dot-Majorana wire system

**DOI:** 10.1038/s41598-024-66478-z

**Published:** 2024-08-01

**Authors:** Piotr Majek, Ireneusz Weymann

**Affiliations:** Institute of Spintronics and Quantum Information, Faculty of Physics, Adam Mickiewicz University, ul. Uniwersytetu Poznańskiego 2, 61-614 Poznań, Poland

**Keywords:** Nanoscale devices, Ferromagnetism, Spintronics, Electronic and spintronic devices

## Abstract

In this work we investigate the spin-dependent transport through a double quantum dot embedded in a ferromagnetic tunnel junction and side attached to a topological superconducting nanowire hosting Majorana zero-energy modes. We focus on the transport regime when the Majorana mode leaks into the double quantum dot competing with the two-stage Kondo effect and the ferromagnetic-contact-induced exchange field. In particular, we determine the system’s spectral properties and analyze the temperature dependence of the spin-resolved linear conductance by means of the numerical renormalization group method. Our study reveals unique signatures of the interplay between the spin-resolved tunneling, the Kondo effect and the Majorana modes, which are visible in the transport characteristics. In particular, we uncover a competing character of the coupling to topological superconductor and that to ferromagnetic leads, which can be observed already for very low spin polarization of the electrodes. This is signaled by an almost complete quenching of the conductance in one of the spin channels which is revealed through perfect conductance spin polarization. Moreover, we show that the conductance spin polarization can change sign depending on the magnitude of spin imbalance in the leads and strength of interaction with topological wire. Thus, our work demonstrates that even minuscule spin polarization of tunneling processes can have large impact on the transport properties of the system.

## Introduction

The exploitation of spin degrees of freedom in studies of transport through nanostructures has opened new avenues for exploring a plethora of different many-body phenomena where the interplay of spin and charge determines the system’s transport behavior^1–6^. Interestingly, the spin-selective investigations have allowed one to explore various bound states, including topologically protected states, such as the Majorana zero-energy modes^7–18^. The Majorana bound states are solid-state realizations of Majorana fermions, i.e., particles that are their own anti-particles^19^. The Majorana modes have been predicted to appear in a topologically nontrivial phase of a spinless tight-binding chain with superconducting pairing of the same spin species between neighboring sites, i.e., in the so-called Kitaev chain^20^. Experimentally, such a chain, also referred to as a Majorana wire, can be implemented by placing a semiconducting nanowire with strong spin-orbit interaction in proximity with an *s*-wave superconductor in the presence of external magnetic field^21,22^, or in a chain of magnetic adatoms on superconducting substrate^23–25^. Moreover, Kitaev chains could be also implemented in the absence of external magnetic fields, by exploiting spiral spin structure of the chain atoms^26^. Very recently, a minimal Kitaev chain based on coupled quantum dots has also been implemented^27–29^.

Although a great experimental endeavor has already been taken^30–34^, which has been stimulated by possible applications in fault-tolerant quantum computation^35–37^, a unique and unambiguous confirmation of the existence of Majorana modes is still awaited, despite great recent progress^27,38^. From this perspective, it is important to continue further investigations on transport properties that could provide other indications of the presence of Majorana quasiparticles^39–43^. One of promising ways is to explore the transport characteristics of side-attached zero-dimensional systems, to which topologically protected Majorana modes can leak giving rise to fractional values of the conductance^44–47^. In this respect, there have already been examinations involving both single and multiple quantum dot systems^48–66^. Moreover, the considerations have also concerned the strong coupling regime, where Majorana quasiparticles interact with the Kondo correlations^67–70^. As mentioned above, further information about Majorana modes could be obtained from spin-selective investigations, e.g. by embedding the nanostructure into a ferromagnetic junction or by using spin-polarized spectroscopy. Such considerations have been recently performed for single quantum dots attached to topological superconducting wires^71,72^, while much less is known about the properties of double quantum dot systems, in which the Majorana-Kondo interplay results in further interesting features^73,74^.

**Figure 1:**
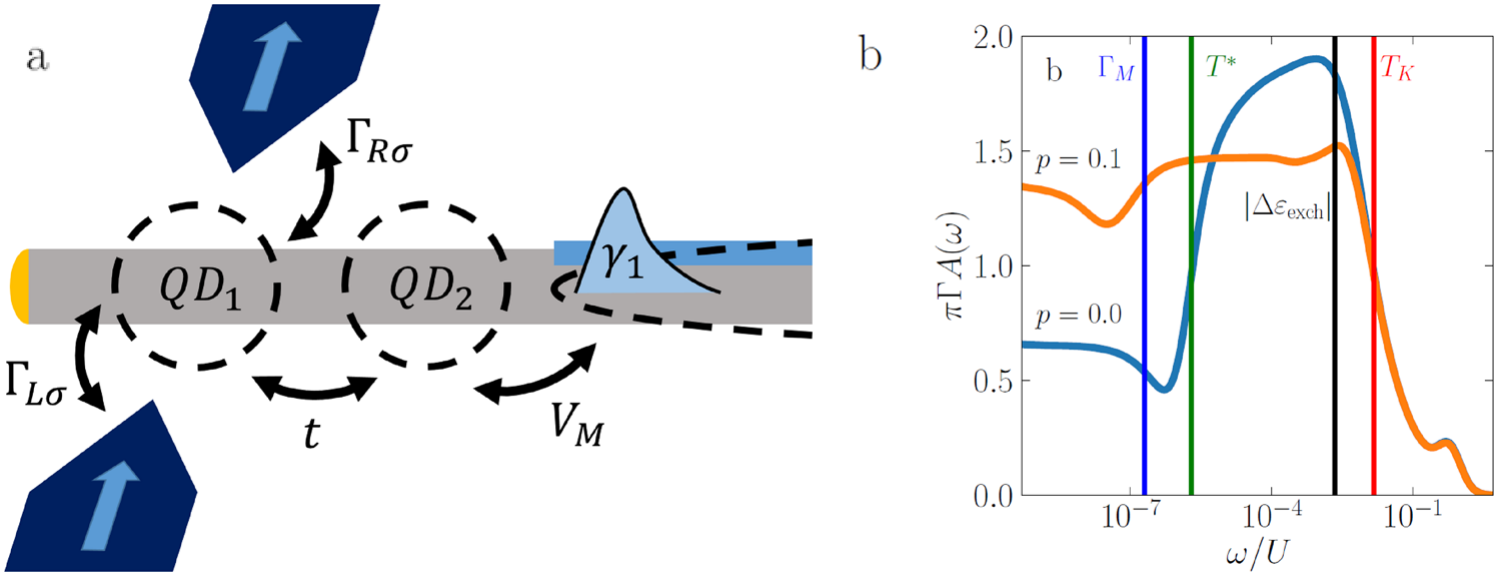
The schematic of the considered double quantum dot-Majorana wire system is presented in the panel (**a**). The first quantum dot is attached to two ferromagnetic leads through the coupling strengths $\Gamma _{j\sigma }$, the two dots are coupled through the hopping matrix elements *t*, while the second quantum dot is connected to a superconducting nanowire in the topological phase through the hopping matrix elements $V_M$. $\gamma _1$ denotes the Majorana zero-energy mode that emerges at the end of the nanowire. We note that our considerations are also relevant for systems with only one ferromagnetic contact. In panel (**b**) we show the energy dependence of the spectral function of the first quantum dot, obtained for a set of representative parameters with $p=0$ and $p=0.1$, where all the relevant energy scales of the model considered (for $p = 0$) are presented. $T_K$ is the (first-stage) Kondo temperature, $T^*$ stands for the second-stage Kondo temperature, $\Gamma _M$ indicates the energy scale associated with the Majorana zero-energy mode, and $|\Delta \varepsilon _{\mathrm{exch}}|$ shows the magnitude of the exchange field. For more details, see the main text.

The goal of this work is therefore to extend the existing studies by addressing the problem of spin-dependent transport properties of a double quantum dot system attached to nanowire hosing Majorana quasiparticles at its ends. In particular, we consider the case when one of the dots is coupled to ferromagnetic electrodes, while the second quantum dot is connected to the Majorana wire forming a T-shaped geometry, as displayed in Fig. 1a. Our considerations are performed with the aid of the numerical renormalization group (NRG) method^75,76^, which allows for taking into account all correlation effects in a fully non-perturbative manner^77^. We have in particular determined the spectral properties of the system for various coupling strengths to the Majorana wire. Moreover, we have analyzed the temperature as well as gate voltage dependence of the spin-resolved linear conductance. These quantities showed features of the interplay between an exchange field, that is triggered by the presence of ferromagnets^78–80^, the Kondo correlations^81,82^ and the Majorana quasiparticles. In particular, we demonstrate that, depending on the strength of coupling to the Majorana wire, the system’s conductance strongly depends on the value of spin polarization of ferromagnetic contacts, and already very low spin polarization is sufficient to drastically modify the conductance of the device. Our work provides thus further insight into the physics of hybrid Majorana wire-quantum dot systems where spin-selective transport plays an important role.

## Methods

### System’s Hamiltonian

The considered system is presented in Fig. 1a. It consists of two quantum dots (QD), arranged in a T-shaped geometry, attached to a topological superconducting nanowire hosting Majorana bound states at its ends. The first quantum dot (QD1) is coupled to the external ferromagnetic leads and the second one (QD2) to the nanowire. The general Hamiltonian that models the system can be written as $H = H_\mathrm{leads} + H_\mathrm{tun} + H_\mathrm{DD-Maj}$. The first part,

1 $$\begin{aligned} H_\mathrm{leads} = \sum _{r=L,R}\sum _{\textbf{k}\sigma } \varepsilon _{r\textbf{k}\sigma } c^\dag _{r\textbf{k}\sigma } c_{r\textbf{k}\sigma }, \end{aligned}$$

describes the leads as a source of non-interacting electrons with energy $\varepsilon _{r\textbf{k}\sigma }$ in the left and right ($r = L, R$, respectively) electrode. The operator $c^\dag _{r\textbf{k}\sigma }$ creates an electron with spin $\sigma = \uparrow , \downarrow$ and momentum $\textbf{k}$ in the *r*-th electrode. The next part of the Hamiltonian models the tunneling between the first quantum dot and the electrodes

2 $$\begin{aligned} H_\mathrm{tun} = \sum _{r=L,R}\sum _{\textbf{k}\sigma } v_{r \sigma } \left( d^\dag _{1\sigma } c_{r\textbf{k}\sigma } + c^\dag _{r\textbf{k}\sigma } d_{1\sigma } \right) , \end{aligned}$$

where $v_{r \sigma }$ describes the momentum independent tunnel matrix elements, and $d^\dag _{1\sigma }$ creates an electron with spin $\sigma$ on the first quantum dot, which is directly attached to the ferromagnetic leads. The last term of the Hamiltonian models effectively the double quantum dot system coupled to the superconducting topological nanowire. It can be written as^48,83^

3 $$\begin{aligned} H_\mathrm{DD-Maj}=\sum _{j=1,2}\sum _{\sigma } \varepsilon _j d_{j\sigma }^\dag d_{j\sigma } + U \sum _{j=1,2} d_{j\uparrow }^\dag d_{j\uparrow } d_{j\downarrow }^\dag d_{j\downarrow } + t \sum _\sigma (d_{1\sigma }^\dag d_{2\sigma } + d_{2\sigma }^\dag d_{1\sigma }) + \sqrt{2} V_M (d^\dag _{2\downarrow } \gamma _1 + \gamma _1 d_{2\downarrow }), \end{aligned}$$

where the first two terms describe the first and second ($j = 1, 2$) quantum dot with energy $\varepsilon _j$ and Coulomb correlations *U*, assumed to be equal for both dots, respectively. The matrix element *t* stands for the hopping between the dots. The last part couples the spin-down electrons on the second quantum dot with the Majorana bound state $\gamma _1$ at the end of the topological superconducting nanowire, with $V_M$ being the relevant tunnel matrix elements. Here, we assume that the wire is sufficiently long, such that the overlap between the Majorana modes at both ends of the nanowire is negligible. The broadening of the first quantum dot energy level due to the coupling to ferromagnetic leads is given by $\Gamma _{r \sigma } = \pi \rho _{r \sigma } v_{r \sigma }^2$, where $\rho _{r \sigma }$ stands for the density of states at the Fermi level of the lead *r* for spin $\sigma$. This coupling can be rewritten as $\Gamma _{r \sigma } = (1 + \sigma p_r) \Gamma _r$, with $\Gamma _r = (\Gamma _{r \uparrow } + \Gamma _{r \downarrow })/2$ and $p_r$ referring to the spin polarization of the lead *r*. We assume a flat density of states of the leads and use its half-width as the energy unit, $D \equiv 1$. Moreover, for the sake of simplicity of further analysis, we assume that the quantization axis coincides with the Majorana polarization and that Majorana mode couples to spin-down electrons on the second dot^68,71,73^. We also assume the magnatizations of the leads point in the same direction, i.e. the system is in the parallel magnetic configuration. In this work we are interested in the linear response transport properties.

It is then convenient to perform an orthogonal left-right transformation to a new basis by introducing the even linear combination of the left and right electron operators as follows

$$\begin{aligned} a_{\textbf{k}\sigma } = \frac{1}{\sqrt{v_{L \sigma }^2+v_{R \sigma }^2}} (v_{L \sigma }c_{L\textbf{k}\sigma } + v_{R \sigma }c_{R\textbf{k}\sigma }). \end{aligned}$$

After such transformation, the first quantum dot couples to one effective electronic reservoir, with a new coupling strength $\Gamma = \Gamma _L + \Gamma _R$ and an average spin polarization $p=(p_L + p_R)/2$^84^. We note that this transformation is very general and also holds when one of the couplings vanishes or one of the spin polarizations is zero, i.e., when there is only one lead in the system or when one of the contacts is nonmagnetic. Consequently, our considerations shall be relevant for other geometries as well, e.g., the ones involving only one ferromagnetic contact, which could be a substrate or a spin-polarized tip of STM.

### Computation method

The calculations are performed with the aid of the numerical renormalization group method involving the full density matrix^76,77,85^. The clue of the NRG method is a logarithmic discretization of the conduction band and mapping of such discrete Hamitlonian to a tight-binding chain with exponentially decaying hopping integrals $\xi _n$^75^. In our case the NRG Hamiltonian has the following form

4 $$\begin{aligned} H = H_\mathrm{DD-Maj} + \sum _{\sigma } \sqrt{\frac{2\Gamma _\sigma }{\pi }} \left( d^\dag _{1\sigma } f_{0\sigma } + f^\dag _{0\sigma } d_{1\sigma } \right) +\sum _{n,\sigma } \xi _n \left( f^\dag _{n\sigma } f_{n+1\sigma } + f^\dag _{n+1\sigma } f_{n\sigma } \right) , \end{aligned}$$

where $f_{n\sigma }$ is the annihilation operator of an electron with spin $\sigma$ on the *n*th site of the chain, with the zeroth-site operator given by $f_{0\sigma } = {\mathscr {N}}^{-1/2} \sum _\textbf{k} a_{\textbf{k}\sigma }$. This Hamiltonian can be solved iteratively by keeping an appropriate number of low-energy eigenstates during the iteration $N_{\mathrm{kept}}$. In our computations we keep at least $N_{\mathrm{kept}} = 4000$ states. We also use the logarithmic discretization parameter $\Lambda = 2$. The spectral data is determined in the Lehmann representation and collected in logarithmic bins that are then broadened to obtain smooth functions. On the other hand, the conductance is obtained directly from the discrete data without the need to introduce broadening^86^. In calculations, we make use of the conservation of spin-up particles and also exploit the parity symmetry of the Hamiltonian.

## Results and discussion

### Relevant energy scales

The transport properties of the system are conditioned by relative interplay of various energy scales. One of such scales, referred to as the exchange field, is set by the spin-resolved charge fluctuations between the first quantum dot and ferromagnetic contacts. Such exchange field can be estimated from the second-order perturbation theory. Assuming singly-occupied quantum dot, one can define the spin splitting as $\Delta \varepsilon \equiv \delta \varepsilon _\uparrow - \delta \varepsilon _\downarrow$, where $\delta \varepsilon _\sigma$ denotes the second-order correction to the energy level due to spin-resolved charge fluctuations between the state $|\sigma \rangle$ and the empty or fully occupied states, $|0\rangle$ and $|2\rangle$, respectively. In the case of zero temperature and vanishing hopping between the dots, $t \rightarrow 0$, one finds^78^

5 $$\begin{aligned} \Delta \varepsilon _{\mathrm{exch}}\approx \frac{2p \Gamma }{\pi } \log \left| \frac{\varepsilon _1}{\varepsilon _1+U}\right| . \end{aligned}$$

As can be seen from the above formula, $\Delta \varepsilon _{\mathrm{exch}}$ vanishes for $\varepsilon _1 = -U/2$. Thus, finite splitting of the dot level occurs only when there is a detuning from the particle-hole symmetry point. Another spin splitting can be also induced by the coupling to topological superconductor, which can split the levels of the second quantum dot. The magnitude of this splitting in the leading order in $V_M$ and in the local moment regime of the second quantum dot can be found from the eigenenergies of the effective low-energy Hamiltonian^68,71^

6 $$\begin{aligned} \Delta \varepsilon _{\mathrm{M}}\approx \frac{2V_M^2}{U^2} \left( 2\varepsilon _2+U\right) . \end{aligned}$$

Again, this splitting is only finite for $\varepsilon _2\ne -U/2$. Nevertheless, it is important to note that even for $\varepsilon _2= -U/2$, the presence of Majorana quasiparticles can give rise to quantum interference effects that could result in fractional values of the conductance^68^. The relevant energy scale associated with such Majorana interference will be denoted by $\Gamma _M$, and it can be defined as $\Gamma _M= \pi \rho _{QD_1} V_M^2$, where $\rho _{QD_1}$ denotes the density of states of the first quantum dot^68^.

We also note that the above formulas for the corresponding spin splittings are only approximate and one needs to keep in mind that, due to the hopping between the two dots, both splittings will vanish only when the system is fully at the particle-hole symmetry point, i.e. when $\varepsilon _1 = \varepsilon _2 = -U/2$.

Further important energy scales are established by the strong electron correlations driving the Kondo effect. In T-shaped double quantum dots a two-stage Kondo effect can emerge^87–92^. In this type of the Kondo phenomenon, with lowering the temperature *T*, the spin of the first quantum dot becomes screened when $$T<T_K$$, giving rise to an enhanced conductance. However, further decrease in *T*, gives rise to screening of the spin of the second quantum dot, which happens for $$T<T^*$$, where $$T^*$$ denotes the second-stage Kondo temperature. This happens due to non-zero hopping *t* between the dots, which gives rise to the inter-dot exchange coupling. Then, since the second dot is not directly coupled to the leads, the conductance becomes suppressed. The reason for such behavior is that the first QD forms a Fermi liquid with the leads, while the second quantum dot starts to serve as an impurity, where the resonant scattering results in total suppression of the conductance for temperatures lower than the second-stage Kondo temperature, $$T < T^*$$^87–89^. In the case of ferromagnetic leads, the Kondo effect will develop in the system only when the Kondo temperature $$T_K$$ is larger than the exchange field, $$T_K> |\Delta \varepsilon _{\mathrm{exch}}|$$, which will happen around $$\varepsilon _1\approx -U/2$$. Thus, $$T_K$$ for $$\varepsilon _1 = -U/2$$ could be estimated from^78,93^

7 $$\begin{aligned} T_K\approx \sqrt{\frac{U\Gamma }{2}}\exp \left[ -\frac{\pi U \;\mathrm{arctanh} (p)}{8\Gamma \; p} \right] . \end{aligned}$$

On the other hand, the second-stage Kondo temperature at the particle-hole symmetry point can be found from^87,89^

8 $$\begin{aligned} T^* \approx a \; T_K \; \exp \left( \! -b \; \frac{ T_K U}{4t^2}\right) , \end{aligned}$$

where *a* and *b* are constants of the order of unity^94^. All the relevant energy scales introduced above are marked explicitly in Fig. 1b, which shows the energy dependence of the local density of states of the first quantum dot.

### Spectral properties

**Figure 2:**
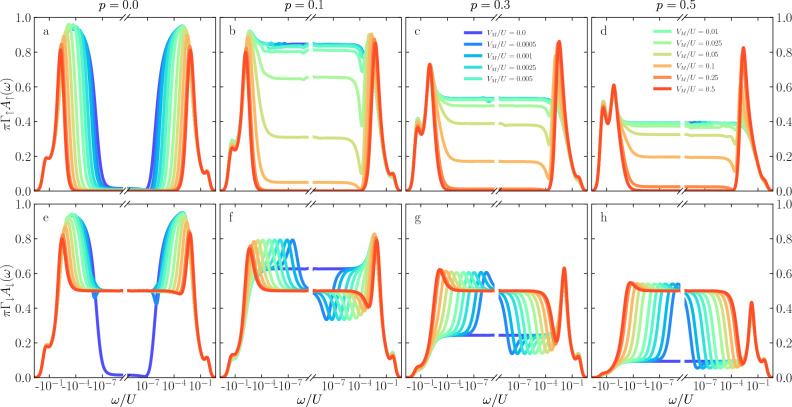
The normalized spin-resolved spectral functions of the first quantum dot plotted as a function of energy $\omega$ on the logarithmic scale. The top row (**a**–**d**) presents the spin-up contribution, while the bottom row (**e**–**h**) shows the spin-down contribution to the spectral function calculated for different values of the coupling to the topological superconductor $V_M$, as indicated in the legend. The consecutive columns present the results for different values of the spin polarization of the leads *p*, as indicated. The other parameters are as follows: $U=0.2$, $\Gamma =0.02$, $t=0.004$, in units of band half-width, and $T=0$. The quantum dots’ orbital levels are set as $\varepsilon _{1} = -U/3$ and $\varepsilon _{2} = -U/2$.

Before analyzing the system’s conductance, it is important to discuss the behavior of the spin-resolved spectral functions that will determine the transport characteristics. The spin-resolved spectral function of the *j*th quantum dot is defined as ${A_{j\sigma } (\omega ) = - (1/\pi ) \;\mathrm{Im} \langle \!\langle d_{j \sigma }|d^{\dagger }_{j \sigma }\rangle \!\rangle _\omega ^\mathrm{ret}}$, where $\langle \!\langle d_{j \sigma }|d^{\dagger }_{j \sigma }\rangle \!\rangle _\omega ^\mathrm{ret}$ is the Fourier transform of the retarded Green’s function of the corresponding quantum dot. In the following, we will focus on the behavior of $A_{1\sigma } (\omega ) \equiv A_{\sigma } (\omega )$, since it determines the system’s conductance in the considered geometry, see Fig. 1a. However, for the sake of completeness of this analysis, we also present the behavior of the spin-resolved spectral functions of the second quantum dot. The corresponding results are presented and discussed in the Appendix. We also notice that the case of fully particle-hole symmetric model would essentially correspond to a nonmagnetic system with modified Kondo temperature^73^. Therefore, we will consider the cases when at least one of the quantum dots’ energy levels is detuned from its particle-hole symmetry point, such that there is always finite splitting associated either with Majorana mode or with proximity to ferromagnetic leads.

Figure 2 presents the energy dependence of the spin-resolved normalized spectral function of the first quantum dot, $\pi \Gamma _\sigma A_\sigma (\omega )$. To resolve the relevant energy scales, the spectral functions are plotted on a logarithmic scale, and we consider the case of zero temperature. The top row displays the spin-up component, while the bottom row shows the spin-down contribution to the spectral function. Each curve is calculated for different coupling $V_M$ to the superconducting nanowire, as indicated in the legend, while each column refers to a different value of the spin polarization of the leads *p*, as marked in the figure. This figure is calculated for $\varepsilon _{1} = -U/3$ and $\varepsilon _{2} = -U/2$, such that $\Delta \varepsilon _{\mathrm{exch}}\ne 0$, while $\Delta \varepsilon _{\mathrm{M}}\approx 0$.

The first column presents the reference case when $p = 0$, i.e., when the electrodes are nonmagnetic. Here, one can distinguish three different energy scales: the ones associated with the first and the second stage of the Kondo screening, $T_K$ and $T^*$, respectively, and the third one related to the quantum interference with the Majorana wire, denoted by $\Gamma _M$. As can be seen in the figure, for $V_M=0$, the spectral function exhibits two resonances around $\omega \approx \pm T_K$ due to the first-stage Kondo effect, while for $|\omega | \lesssim T^*$, $A_{\sigma }(\omega )$ becomes suppressed due to the second-stage of Kondo screening. Finite coupling to Majorana wire, generally blocks the second-stage screening in the spin-down channel, giving rise to $\pi \Gamma _\downarrow A_\downarrow (0) = 1/2$. The range of energies around the Fermi level where $\pi \Gamma _\uparrow A_\uparrow (\omega ) = 0$ while $\pi \Gamma _\downarrow A_\downarrow (\omega ) = 1/2$ grows with increasing $V_M$, which indicates that the presence of Majorana mode alters the Kondo correlations down to energy scales of $\omega \approx \Gamma _M$. In other words, finite $V_M$ enhances the second stage Kondo temperature. We note that qualitatively similar results are obtained even for finite *p* when $\varepsilon _{1} = \varepsilon _{2} = -U/2$.

**Figure 3:**
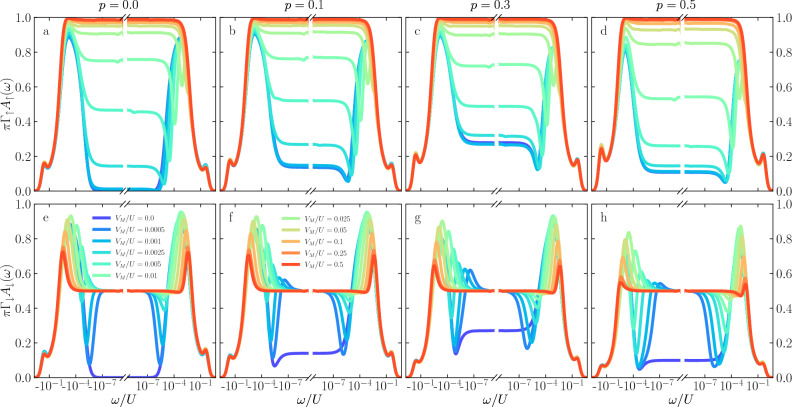
The normalized spin-resolved spectral functions of the first quantum dot calculated for $\varepsilon _{1} = -U/2$ and $\varepsilon _{2} = -U/3$. The other parameters are the same as in Fig. 2.

**Figure 4:**
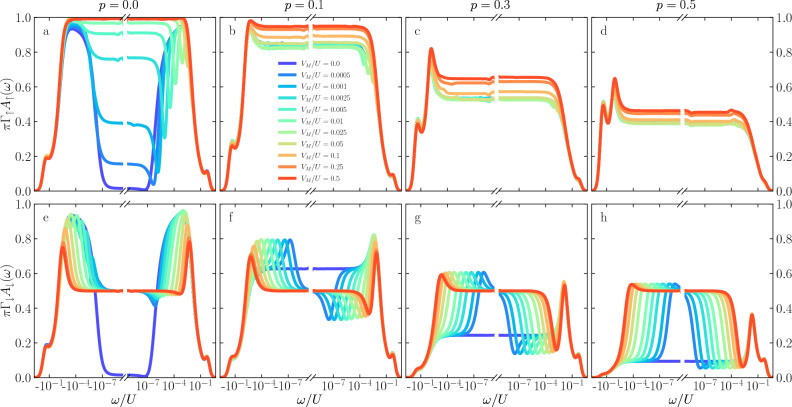
The normalized spin-resolved spectral functions of the first quantum dot calculated for $\varepsilon _{1} = \varepsilon _{2} = -U/3$ and the other parameters as in Fig. 2.

The cases of finite spin polarization are shown in the next columns of Fig. 2, where the second column is calculated for $p=0.1$, the third one for $p=0.3$, while the last one displays the case of $p=0.5$. Note that this sequence also applies to Figs. 3 and 4. As can be seen, having non-zero leads polarization *p*, which results in finite exchange field $\Delta \varepsilon _{\mathrm{exch}}$, significantly modifies the whole picture discussed above for both spin contributions. When the spin polarization is introduced, the majority contribution is associated with the spin-up electrons. As can be seen in Fig. 2b, this suppresses the second stage of Kondo screening for $V_M = 0$, restoring the low energy transport. This happens since $T^*\lesssim |\Delta \varepsilon _{\mathrm{exch}}| \lesssim T_K$. Increasing the coupling to the Majorana wire, the influence of the ferromagnetic leads is being reduced (since $T^*$ grows with $V_M$), and the system demonstrates the two-stage Kondo effect again once $V_M\gtrsim 0.1\;U$, resulting in $A_\uparrow (0) = 0$. One can also notice a dip that occurs for $\omega \approx 10^{-4}\;U$, which deepens as the coupling to topological superconductor reaches $V_M=0.01\;U$. This deep is an indication of the presence of exchange field splitting of the first quantum dot level. On the other hand, the spin-down normalized spectral function $\pi \Gamma _\downarrow A_\downarrow (\omega )$ for $p=0.1$ is shown in Fig. 2f. As for the spin-up contribution, when there is no coupling to the Majorana wire, the presence of ferromagnetic leads affects the spin screening on the second quantum dot, what results in destroying the second-stage of the Kondo effect. However, when the Majorana coupling is present in the system, it immediately restores the low-energy spectral function to the value of $\pi \Gamma _\downarrow A_\downarrow (0) = 1/2$. Interestingly, one can also observe asymmetric peaks and dips, which maximum (minimum) shifts with the increasing value of coupling to Majorana wire. These features occur at energies corresponding to $\Gamma _M$.

This picture is modified for the higher values of the leads polarization, as shown in further columns of Fig. 2. Generally, one observes a gradual suppression of the low-energy spectral functions in both spin components. This is due to the fact that for the considered values of *p*, $T_K\lesssim |\Delta \varepsilon _{\mathrm{exch}}|$ for $V_M=0$, such that also the first stage Kondo effect becomes suppressed. Nonetheless, for finite coupling to Majorana wire, one can observe a restoration of the second-stage screening, which results in $A_\uparrow (0) = 0$, while for the spin-down component one again finds, $\pi \Gamma _\downarrow A_\downarrow (0) = 1/2$. What also draws the attention here is the suppression of asymmetric minima and maxima visible in $A_\downarrow (\omega )$ with increasing *p*. When the exchange field is considerable, see Fig. 2h, one basically observes a restored resonance at the Fermi level with $\pi \Gamma _\downarrow A_\downarrow (0) = 1/2$.

The case when the quantum dots’ orbital levels are tuned to $\varepsilon _{1} = -U/2$ and $\varepsilon _{2} = -U/3$ is shown in Fig. 3. (Note that this figure is calculated for the same set of parameters as Fig. 2.) This corresponds to the situation when the ferromagnet-induced exchange field is negligible $\Delta \varepsilon _{\mathrm{exch}}\approx 0$, while there is a finite splitting caused by the coupling to the Majorana wire, $\Delta \varepsilon _{\mathrm{M}}\ne 0$. In the first column, the case of nonmagnetic leads is presented. Contrary to the situation shown in Fig. 2, increasing the coupling to the topological superconductor, the Kondo peak starts to form, reaching its maximum when $V_M \gtrsim 0.1\;U$. The spin-down spectral function exhibits a weaker influence on the coupling to the Majorana wire than it was shown in Fig. 2. One can notice that the low-energy spectral function is restored to the half of its maximum, $\pi \Gamma _\downarrow A_{\downarrow } (0) = 1/2$, however, this change is not as immediate as it was in the case shown in Fig. 2e. With increasing the spin polarization *p*, the value of the spectral function at the Fermi level for negligible $V_M$ starts increasing until $p=0.3$, to drop when $p=0.5$. Such a nonmonotonic behavior can be explained by realizing that the presence of ferromagnetic leads also affects the singlet and triplet states of the double quantum dot. Once the energy of one of the components of the triplet becomes comparable with the energy of the singlet state, which can happen for certain value of *p*, the low-energy spectral function becomes enhanced due to Kondo processes^92^. One can also see that the presence of the coupling to topological superconductor restores the fractional value of $\pi \Gamma _\downarrow A_{\downarrow } (0)$ irrespective of *p*. On the other hand, the spin-up component for large enough $V_M$ displays the Kondo resonance at the Fermi energy, which hardly depends on *p*. This is associated with the fact that, for $\varepsilon _2 = -U/3$, coupling to the Majorana wire induces a considerable spin splitting of the second quantum dot level, such that the second-stage Kondo screening becomes then negligible. Note also that the quantum interference peaks and dips visible in Fig. 2 are now suppressed, since the presence of Majorana quasiparticles is mainly revealed through the splitting $\Delta \varepsilon _{\mathrm{M}}$.

Finally, let us discuss the behavior of the spectral functions when the orbital levels of both quantum dots are set to $\varepsilon _{1} = \varepsilon _{2} = - U/3$. This case is presented in Fig. 4. In this figure one can generally recognize the combination of the effects that are visible in Figs. 2 and 3. This is because now we have both the spin splitting due to the ferromagnetic exchange field as well as the splitting caused by the coupling to the Majorana wire. Interestingly, one can see that the behavior of the spin-up spectral function somewhat resembles the dependence presented in the first row of Fig. 2. On the other hand, the behavior of the spin-down spectral function bears some similarity to the corresponding spectral function shown in Fig. 3. This can be understood by realizing that the spin-down component is greatly affected by the presence of Majorana quasiparticles, so the results are similar when Majorana splitting is finite, i.e. in the case of $\varepsilon _2 = -U/3$. For the spin-up component it is the exchange field that is the most relevant, so now the results for $\varepsilon _1=-U/3$ bear the corresponding resemblance. Of course, this analysis is somewhat superficial and a closer inspection reveals crucial differences. In particular, it can be seen that for finite *p* the normalized spectral function for spin-up electrons, $\pi \Gamma _\uparrow A_\uparrow (\omega )$, becomes less vulnerable to the influence of the coupling to the topological superconductor, and is more fixed by the leads spin polarization. This can be explained by the fact that increasing *p* induces larger $\Delta \varepsilon _{\mathrm{exch}}$, which can partly suppress the first-stage Kondo effect. Indeed, the value of the spectral function at the Fermi level decreases, while a pronounced split Kondo peak becomes visible for negative energies. Moreover, since the quantum interference with the Majorana wire is suppressed by finite splitting of the second dot level due to $\Delta \varepsilon _{\mathrm{M}}$, changing $V_M$ has a rather weak influence on $A_\uparrow (\omega )$. Consequently, now the second-stage Kondo effect cannot be restored, see Figs. 4b–d.

As far as the behavior of the spin-down spectral function is concerned, one can see much larger dependence on the coupling to the Majorana wire. For $V_M=0$, increasing *p* results in a stronger suppression of $A_\downarrow (0)$, while a split Kondo peak develops for positive energies. However, even small values of $V_M$ change the picture drastically by bringing the spectral function at the Fermi energy to the fractional value of $\pi \Gamma _\downarrow A_\downarrow (0)=1/2$. Moreover, while for $p=0.1$ there are clear maxima (minima) for $\omega <0$ ($\omega >0$) indicating interference in the spin-down channel, with increasing *p* these features become suppressed. In fact, for $p=0.5$, there is a pronounced zero-energy peak of height $\pi \Gamma _\downarrow A_\downarrow (0)=1/2$, the width of which increases with $V_M$, see Fig. 4h.

### Transport characteristics

**Figure 5:**
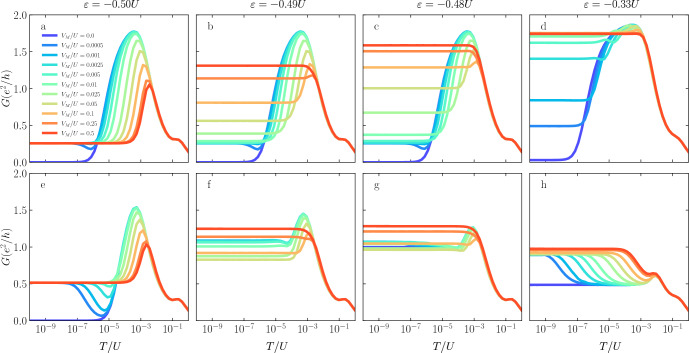
The temperature dependence of the linear conductance for different values of the coupling to Majorana wire, as indicated. The first (second) row presents the data for $p=0$ ($p=0.5$), while each column corresponds to different value of double dot level positions, $\varepsilon \equiv \varepsilon _1=\varepsilon _2$, increasing the detuning from the particle-hole symmetry point, while moving to the right. The other parameters are the same as in Fig. 4.

Let us now focus on the behavior of the linear conductance of the system. The linear conductance in the spin channel $\sigma$ can be found from

9 $$\begin{aligned} G_\sigma = \frac{e^2}{h} \pi \Gamma _\sigma \int d\omega \; A_\sigma (\omega ) \left( -\frac{\partial f(\omega )}{\partial \omega }\right) , \end{aligned}$$

where $f(\omega )$ is the Fermi-Dirac distribution function. The total conductance is given by $G = G_\uparrow + G_\downarrow$, and we can also define the conductance spin polarization as

10 $$\begin{aligned} \mathscr {P}= \frac{G_\uparrow - G_\downarrow }{G_\uparrow + G_\downarrow }. \end{aligned}$$

#### Temperature dependence

Figure 5 presents the temperature dependence of the linear conductance for different values of the coupling to the Majorana wire and for different detuning from the particle-hole symmetry point, $\varepsilon \equiv \varepsilon _1 = \varepsilon _2 =U/2$. The first row, for reference, presents the data in the case of $p=0$, while the second row displays the results for $p=0.5$. In the case of nonmagnetic leads, for $\varepsilon =-U/2$, one can see that increasing $V_M$ destroys the second stage Kondo screening and leads to $G(T<T^*)=e^2/2h$. Moreover, one can see that the larger $V_M$ is, the larger $T^*$ becomes. For finite detuning, increasing $V_M$ again suppresses the second-stage of the Kondo effect, however, now the low-temperature conductance acquires a larger value, which increases with detuning the system more out of the particle-hole symmetry point. The case of ferromagnetic leads and $\varepsilon = -U/2$ is qualitatively similar to the case of $p=0$. The only difference is associated with lower Kondo temperature in the case of finite *p*, thus the maximum value of the conductance for temperatures between $T^*$ and $T_K$ is smaller. On the other hand, when there is a detuning from the particle-hole symmetry point, the exchange field comes into play suppressing the second-stage of the Kondo effect. One then finds a finite low-temperature conductance with small dependence on the value of $V_M$. This dependence becomes however considerable for $\varepsilon =-U/3$ when a nonmonotonic dependence of *G*(*T*) develops, with an enhancement of conductance around $T\sim \Gamma _M$, see Fig. 5h.

**Figure 6:**
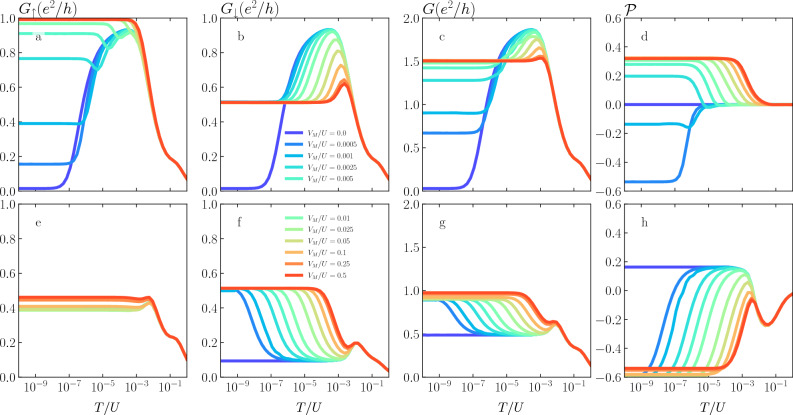
The spin-resolved conductance for the nonmagnetic (first row) and ferromagnetic leads with $p=0.5$ (second row) in the case of $\varepsilon _1=\varepsilon _2=-U/3$. The first (second) column shows $G_\uparrow$ ($G_\downarrow$), the third column presents *G*, while the last column displays $\mathscr {P}$. The other parameters are the same as in Fig. 4.

Let us now inspect the temperature behavior of the spin-resolved conductance and its polarization for $\varepsilon = -U/3$, again for the nonmagnetic and ferromagnetic lead case. This is presented in Fig. 6. The first (second) column presents $G_{\uparrow }$ ($G_\downarrow$), the third column displays *G*, while the last column shows $\mathscr {P}$ as a function of *T*. In this figure one can clearly identify various energy scales determining the system’s behavior. In the case of $p=0$, increasing the coupling to Majorana wire results in suppression of the second stage of screening, such that at low temperatures $G_\uparrow$ approaches $e^2/h$, while $G_\downarrow = e^2/2h$, yielding $G = 3e^2/2h$. A particular dependence on $V_M$ of the spin components of the conductance is also revealed in the behavior of $\mathscr {P}$. For low $V_M$, the conductance spin polarization is negative at low temperatures, while with increasing $V_M$, it changes sign and becomes positive. This is due to the fact that while $G_\downarrow$ increases up to the fractional value of $e^2/2h$ with $V_M$, $G_\uparrow$ grows up to the maximum value, see the first column of Fig. 6. This picture is changed in the case of ferromagnetic leads. First of all, one can see that $G_\uparrow$ only weakly depends on the coupling to the Majorana wire. This can be understood by realizing that it is the spin-down level that is directly coupled to the Majorana wire and the behavior of $G_\uparrow$ is mainly determined by the exchange field splitting. However, this is contrary to the spin-down conductance component, which exhibits a strong dependence on $V_M$. One can see that due to the coupling to Majorana wire, the spin-down conductance becomes enhanced around $T\sim \Gamma _M$. This behavior is reflected in the conductance spin polarization which changes sign and becomes negative at low temperatures in the case of finite coupling to Majorana wire.

#### Interplay of exchange field and Majorana coupling

**Figure 7:**
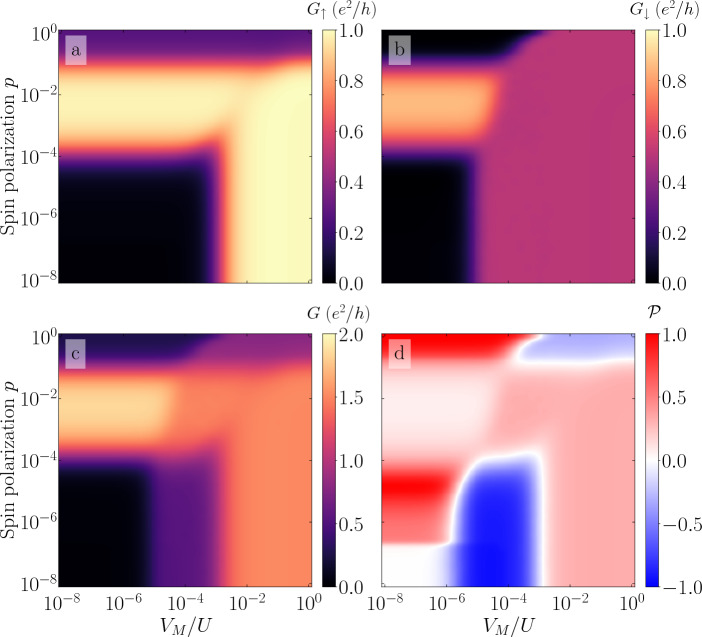
The spin-up (**a**), spin-down (**b**) and total (**c**) linear conductance, as well as the conductance spin polarization (**d**) calculated as a function of the lead spin polarization *p* and the coupling to Majorana wire $V_M$ assuming low temperature and $\varepsilon _1 = \varepsilon _2 = -U/3$. The other parameters are the same as in Fig. 4.

Figure 7 presents the low-temperature spin-resolved conductance, $G_\sigma$ and *G*, together with its spin polarization $\mathscr {P}$, plotted as a function of *p* and $V_M$, calculated for $\varepsilon _1 = \varepsilon _2 = -U/3$ and $T = 5 \times 10^{-10}\; U$, which implies $T\ll T^*$. In this figure one can distinguish significant regimes where the Majorana, Kondo and exchange field physics interplay. For negligible spin polarization of the leads $p\rightarrow 0$, one can see that the total conductance exhibits a monotonic increase with $V_M$. It starts with suppressed conductance $G\approx 0$ for $V_M/U \lesssim 5\times 10^{-6}$ due to the two-stage Kondo effect. For $5\times 10^{-6} \lesssim V_M /U \lesssim 10^{-3}$, one finds $G = e^2/2h$, due to the suppressed second stage of screening in the spin-down channel yielding $G_\downarrow = e^2/2h$. Moreover, when $V_M/ U \gtrsim 10^{-3}$, large coupling to Majorana wire induces $G_\uparrow = e^2/h$, which gives rise to $G = 3e^2/2h$. This behavior is reflected in the dependence of the conductance spin polarization, which is generally positive except for $5\times 10^{-6} \lesssim V_M/U \lesssim 10^{-3}$, where negative spin polarization develops, $\mathscr {P}\approx -1$.

On the other hand, for negligible coupling to Majorana mode, increasing lead spin polarization *p* gives rise to a nonmonotonic dependence of the conductance, which is visible in both spin channels. The mechanism responsible for such nonmonotonicity was already explained earlier and is associated with spin-splitting of the triplet states caused by the exchange field. Moreover, one can see a region of enhanced conductance spin polarization around $p\approx 2\times 10^{-5}$, as well as for large *p*. The enhancement for large *p* is rather intuitive, since then the spin-up electrons dominate transport, which results from high spin asymmetry in the couplings. Such an asymmetry is however not present for *p* as tiny as $p\approx 2\times 10^{-5}$, and the enhanced spin polarization $\mathscr {P}$ is a result of subtle interplay between the spin splitting caused by the exchange field and correlations driving the second stage of the Kondo effect.

Interestingly, the above described behavior extends to finite regions in the *p* and $V_M$ parameter space presented in Fig. 7. For the weak coupling to the Majorana mode and for weakly spin-polarized leads, the conductance is suppressed, which is the manifestation of the second stage Kondo screening. This suppression extends up to $p \approx 10^{-4}$ and $V_M/U \approx 5 \times 10^{-6}$. In this regime, the coupling to Majorana mode as well as the ferromagnet-induced exchange field hardly affect the physics. However, when moving towards stronger values of coupling to Majorana mode and spin polarization, both energy scales start to be relevant. In this regime one can see the competitive character of both $V_M$ and *p* affecting the system’s transport behavior. This competition, although hardly visible in the conductance, is greatly revealed in the dependence of $\mathscr {P}$, which exhibits a sign change along the line $V_M/U \sim p^2$ extending up to $p\approx 10^{-4}$. Further increasing the spin imbalance in the leads, results in $\Delta \varepsilon _{\mathrm{exch}}$ winning over spin polarization caused by the presence of Majorana mode, such that one generally finds $\mathscr {P}>0$. On the other hand, for low *p*, further increasing $V_M$ restores the fractional value of the conductance in the spin-down channel, resulting in maximum negative spin polarization of the conductance, which however again changes sign and becomes positive once $V_M/U \gtrsim 10^{-3}$.

Note also that Fig. 7c nicely presents different fractional values of the conductance extending from $G\approx 0$, through $G\approx e^2/2h$ and $G\approx 3e^2/2h$, up to $G\approx 2 e^2/h$. It can be seen that the corresponding conductance plateau regions not always coincide with the behavior of the conductance spin polarization, which is shown in Fig. 7d. One can then find regimes of full spin polarization, with spin-up or spin-down components dominating transport. When the Majorana coupling suppresses the second stage of the screening in the spin-down channel one finds $\mathscr {P}\approx -1$. However, in the transport regime where the exchange field dominates, $\mathscr {P}$ is enhanced and may reach $\mathscr {P}\approx 1$. On the other hand, for large $V_M$, when the spin-down conductance takes fractional value and the spin-up conductance is maximum, one generally finds $\mathscr {P}\approx 1/3$, irrespective of *p*. Finally, it is important to note that the most interesting physics revealing the interplay between ferromagnetism and coupling to Majorana mode takes place for low values of *p* and $V_M$. Such low values of spin polarization can also result from magnetic impurities or stray fields present in otherwise nonmagnetic electrodes.

## Conclusion

We have determined the spin-resolved transport characteristics of a double quantum dot with ferromagnetic leads side-attached to topological superconducting nanowire hosting Majorana zero-energy modes. The model considered is also relevant for systems with only one ferromagnetic contact, which could be e.g. a tip of a spin-polarized STM. We have shown that the behavior of such system is determined by an intricate interplay between ferromagnet-induced exchange field, quantum interference with the Majorana wire and the Kondo correlations. In fact, for the considered setup, the strong electron correlations can give rise to the first and second-stage Kondo phenomena. To address this interplay in the most reliable manner, our investigations have been carried out with the aid of the non-perturbative numerical renormalization group method. We have in particular determined the spin-resolved spectral functions of the system, together with the linear conductance and its spin polarization for different model parameters, focusing on the transport regimes where various spin correlation effects come into play.

We have shown that the competing character of splittings resulting from ferromagnetic contacts and coupling to the Majorana wire gives rise to unique features in the transport behavior. First of all, both splittings can suppress the second stage of the Kondo screening restoring the conductance to a finite value. In the case of large coupling to topological superconductor, this implies a fractional value of the conductance equal to $3e^2/2h$. On the other hand, in the presence of spin-polarized tunneling, the spectral functions exhibit an antisymmetric resonance at low energies, the position of which depends on the strength of coupling to the Majorana mode. The interplay between the corresponding energy scales is also visible in the behavior of the linear conductance. While the conductance reaches fractional values due to the leakage of the Majorana quasiparticle, spin-polarized tunneling reduces the conductance and it can destroy its well-defined fractional character. Further interesting behavior can be observed in the temperature dependence of the conductance spin polarization, which exhibits a nonmonotonic behavior when tuning the coupling $V_M$ to the Majorana wire, and can change sign depending on the transport regime. This sign change is also nicely visible in the dependence of conductance spin polarization on both the spin imbalance of the leads quantified by *p* and the coupling to topological superconducting wire. We show that even very low values of electrode spin polarization *p* can greatly modify the system’s transport properties giving rise to almost perfect conductance spin polarization, whose sign can be tuned depending on both *p* and $V_M$. It is important to note that such low imbalance in spin-dependent processes could be induced e.g. by stray fields, external magnetic field or presence of magnetic impurities. Our findings provide thus further unique features resulting from the presence of Majorana modes observable in spin-selective transport characteristics.

### Supplementary Information

Supplementary Information.

## Data Availability

The datasets generated and analyzed during the current study are available from the corresponding author on reasonable request.
