# Supplementary material for: Spin-selective transport in a correlated double quantum dot-Majorana wire system
Source: Sci Rep. 2024 Aug 1;14:17762. doi: 10.1038/s41598-024-66478-z (PMC11291930; doi:10.1038/s41598-024-66478-z)
Supplement: Supplementary file 1 — Supplementary Information. [file 41598_2024_66478_MOESM1_ESM.pdf]

Appendix. Spin-selective transport in a correlated double quantum dot-Majorana wire system

In this appendix we present the behavior of the spin-resolved spectral functions of the second quantum dot, i.e., the quantum dot coupled directly to the Majorana wire, calculated for different spin polarizations and couplings to the Majorana mode. Figures 8, 9 and 10 present the results for the same parameters as in Figs. 2, 3 and 4, respectively.

Let us start with the case of $\varepsilon_1 = -U/3$ and $\varepsilon_2 = -U/2$, which is shown in Fig. 8. When $p = 0$, the spectral function displays sharp Hubbard resonances and a pronounced peak at the Fermi energy. The latter peak is a signature of Kondo correlated state developed in the second stage of screening, when the Fermi liquid formed by the conduction band and the first dot screens the spin of the second quantum dot. With increasing V_M, one can see a clear enhancement of the second stage Kondo temperature T^* (the width of the peak at the Fermi energy grows), with asymmetric features developing especially in the case of finite p. Moreover, the effect of spin-polarized tunneling is also visible in the behavior of $A_{2\sigma}(0)$ shown in the insets of Fig. 8. One can observe a suppression of $A_{2\uparrow}(0)$ at low values of V_M compared to $A_{2\downarrow}(0)$. For low spin polarizations, e.g. $p = 0.1$, with increasing V_M the Majorana coupling becomes relevant and consequently $A_{2\uparrow}(0) > A_{2\downarrow}(0)$, see Fig. 8(f) for $V_M/U \approx 0.08$. However, when the lead spin polarization is enhanced further, the exchange field splitting dominates for all considered value of V_M, such that $A_{2\uparrow}(0) < A_{2\downarrow}(0)$, see Fig. 8(h).

The situation when $\varepsilon_1 = -U/2$ and $\varepsilon_2 = -U/3$ is displayed in Fig. 9. Now, $\Delta\varepsilon_{\rm exch}$ is suppressed due to $\varepsilon_1 = -U/2$, while the dominant role is played by $\Delta\varepsilon_{\rm M}$. Because of that, one can see in all the insets of the figure that $A_{2\uparrow}(0) < A_{2\downarrow}(0)$, irrespective of the value of p. Interestingly, there are small asymmetric resonances for low energies visible in $A_{2\downarrow}(\omega)$, which indicate the energy scale associated with the Majorana zero-energy mode. The other qualitative behavior is similar to the previously discussed case – a general enhancement of T^* with increasing V_M is clearly visible.

Finally, the case of both detuned orbital levels, $\varepsilon_1 = \varepsilon_2 = -U/3$, is shown in Fig. 10. In this figure one can recognize a mixture of features discussed in the two previous cases. Furthermore, one now generally finds $A_{2\uparrow}(0) < A_{2\downarrow}(0)$ for all finite values of V_M, with increasing the difference between the two components in the presence of exchange field $\Delta\varepsilon_{\rm exch}$.

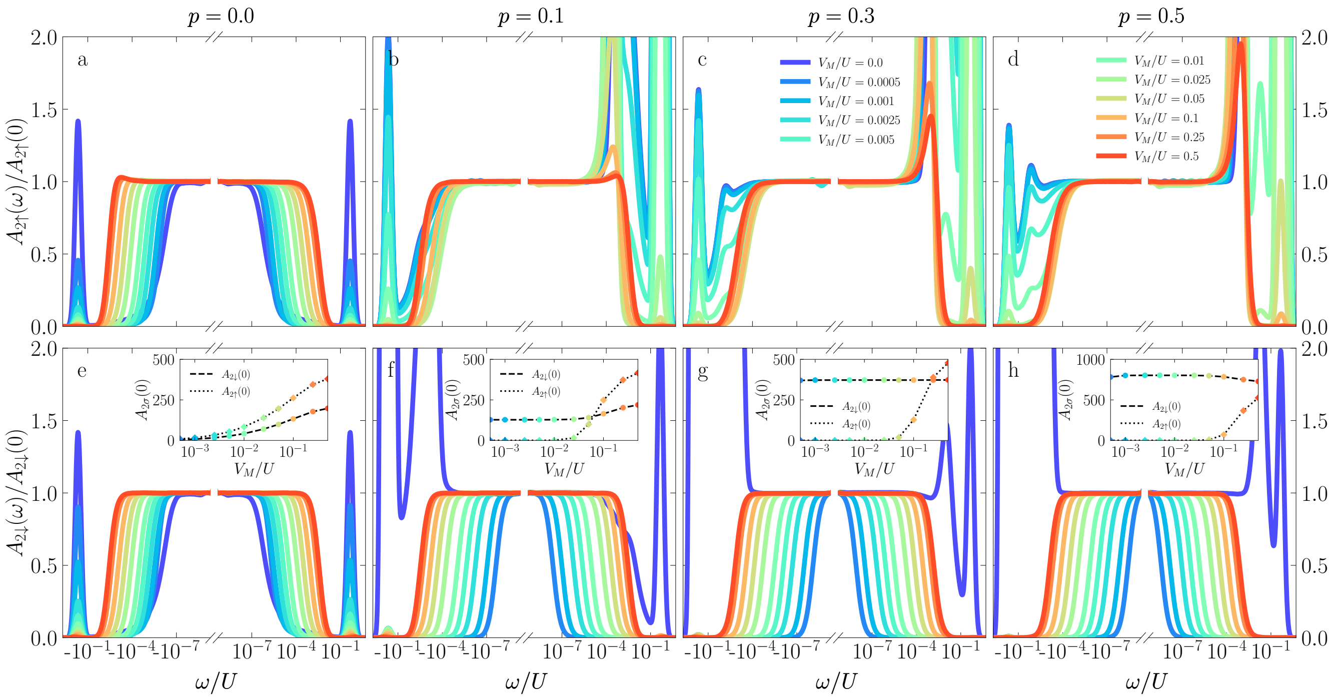

Figure 8. The spin-resolved spectral functions of the second quantum dot normalized to the corresponding value at the Fermi energy for the respective spin component $A_{2\sigma}(0)$, calculated for $\varepsilon_1 = -U/3$ and $\varepsilon_2 = -U/2$. The inset shows the respective values for $A_{2\uparrow}(0)$ (diamonds with dotted line) and $A_{2\downarrow}(0)$ (circles with dashed line) as a function of V_M (colors refer to the corresponding curves). The other parameters are the same as in Fig. 2.

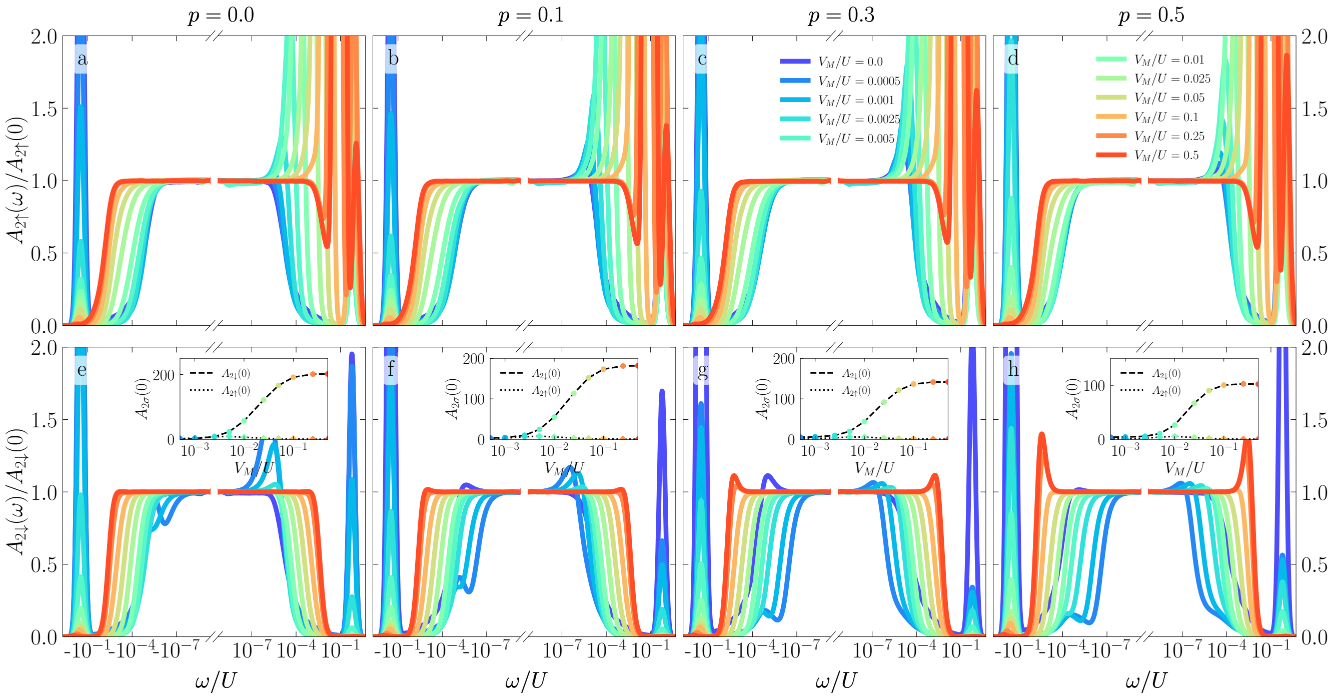

Figure 9. The spin-resolved spectral functions of the second quantum dot normalized to $A_{2\sigma}(0)$, calculated for $\varepsilon_1 = -U/2$ and $\varepsilon_2 = -U/3$. The inset shows the respective values for $A_{2\uparrow}(0)$ (diamonds with dotted line) and $A_{2\downarrow}(0)$ (circles with dashed line) as a function of V_M (colors refer to the corresponding curves). The other parameters are the same as in Fig. 2.

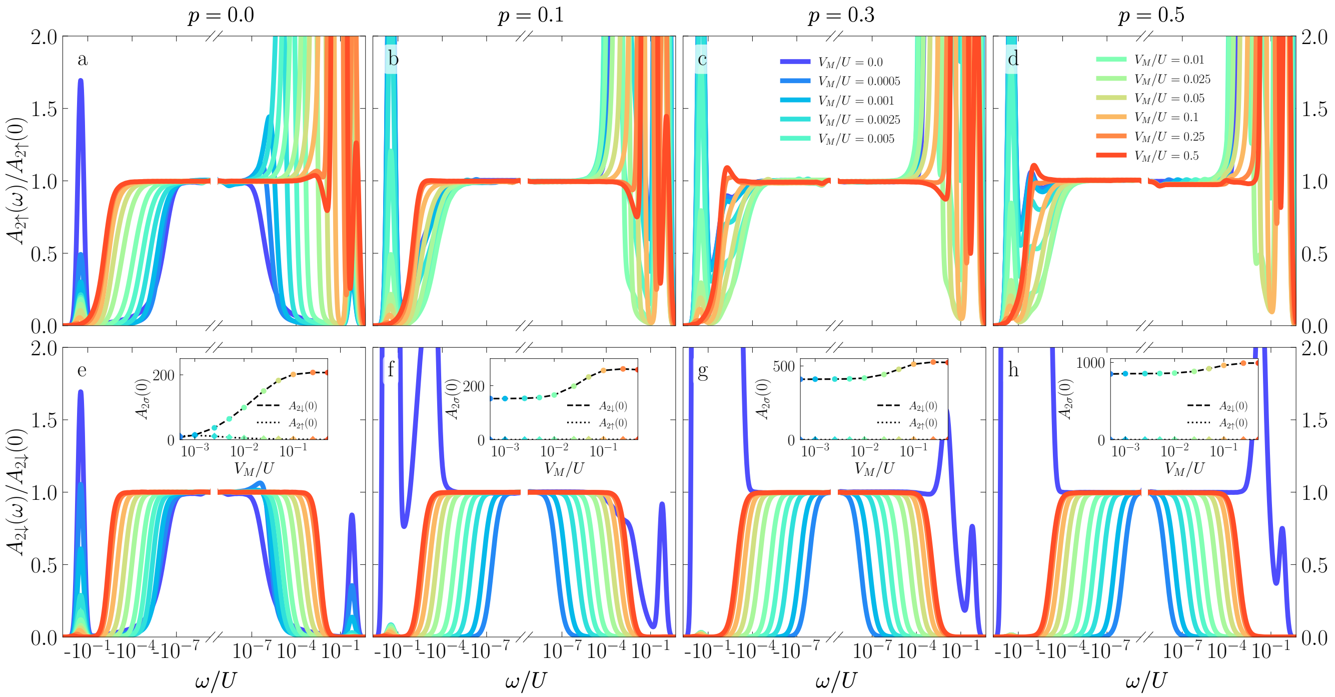

Figure 10. The spin-resolved spectral functions of the second quantum dot normalized to $A_{2\sigma}(0)$, calculated for $\varepsilon_1 = \varepsilon_2 = -U/3$. The inset shows the respective values for $A_{2\uparrow}(0)$ (diamonds with dotted line) and $A_{2\downarrow}(0)$ (circles with dashed line) as a function of V_M (colors refer to the corresponding curves). The other parameters are the same as in Fig. 2.
